# Ligation of Macrophage Fcγ Receptors Recapitulates the Gene Expression Pattern of Vulnerable Human Carotid Plaques

**DOI:** 10.1371/journal.pone.0021803

**Published:** 2011-07-21

**Authors:** Michelle R. Lennartz, Ankur Aggarwal, Tanya M. Michaud, Paul J. Feustel, David M. Jones, M. Julia Brosnan, Rebecca S. Keller, Daniel J. Loegering, Paul B. Kreienberg

**Affiliations:** 1 Center for Cell Biology and Cancer Research, Albany Medical College, Albany, New York, United States of America; 2 Center for Neuropharmacology and Neuroscience, Albany Medical College, Albany, New York, United States of America; 3 Department of Pathology, Albany Medical College, Albany, New York, United States of America; 4 Center for Cardiovascular Sciences, Albany Medical College, Albany, New York, United States of America; 5 Center for Metabolic Disease, Ordway Research Institute, Albany, New York, United States of America; 6 Institute for Vascular Health and Disease, Albany, New York, United States of America; Massachusetts General Hospital and Harvard Medical School, United States of America

## Abstract

Stroke is a leading cause of death in the United States. As ∼60% of strokes result from carotid plaque rupture, elucidating the mechanisms that underlie vulnerability is critical for therapeutic intervention. We tested the hypothesis that stable and vulnerable human plaques differentially express genes associated with matrix degradation. Examination established that femoral, and the distal region of carotid, plaques were histologically stable while the proximal carotid plaque regions were vulnerable. Quantitative RT-PCR was used to compare expression of 22 genes among these tissues. Distal carotid and femoral gene expression was not significantly different, permitting the distal carotid segments to be used as a paired control for their corresponding proximal regions. Analysis of the paired plaques revealed differences in 16 genes that impact plaque stability: matrix metalloproteinases (MMP, higher in vulnerable), MMP modulators (inhibitors: lower, activators: higher in vulnerable), activating Fc receptors (FcγR, higher in vulnerable) and FcγR signaling molecules (higher in vulnerable). Surprisingly, the relative expression of smooth muscle cell and macrophage markers in the three plaque types was not significantly different, suggesting that macrophage distribution and/or activation state correlates with (in)stability. Immunohistochemistry revealed that macrophages and smooth muscle cells localize to distinct and non-overlapping regions in all plaques. MMP protein localized to macrophage-rich regions. *In vitro*, treatment of macrophages with immune complexes, but not oxidized low density lipoprotein, C-reactive protein, or TNF-α, induced a gene expression profile similar to that of the vulnerable plaques. That ligation of FcγR recapitulates the pattern of gene expression in vulnerable plaques suggests that the FcγR → macrophage activation pathway may play a greater role in human plaque vulnerability than previously appreciated.

## Introduction

The morbidity and mortality associated with stroke results from the rupture of vulnerable carotid plaques; plaque removal by carotid endarterectomy significantly decreases stroke risk[1]. In contrast, femoral plaques are histologically and clinically stable, with symptoms resulting from artery occlusion rather than plaque embolism. As stabilizing vulnerable plaques represents a potential therapeutic option, understanding the molecular differences between stable and vulnerable plaques is essential for the design of interventional therapies. Additionally, distinguishing patients with plaques more susceptible to rupture from those that could be managed medically would increase treatment choices for physicians and patients.

A major factor contributing to plaque rupture is degradation of the extracellular matrix. The matrix is predominantly collagen and elastin[2], which can be degraded by matrix metalloproteinases (MMP), specifically collagenase (MMP-1, 8), gelatinase (MMP-9) and elastase (MMP-12). MMPs are proteases, secreted as inactive precursors and activated by proteolytic cleavage, mainly by the plasmin system. Plasmin is generated from plasminogen by urokinase-type plasminogen activator (uPA). When bound to its receptor, uPAR, uPA can produce plasmin for focal activation of MMPs. The proteolytic activity of mature MMPs is regulated by their association with tissue inhibitors of metalloproteinases (TIMPs). Thus, the MMP:TIMP ratio determines overall MMP activity [3].

Vulnerable plaques express elevated levels of MMP-1, MMP-8, and MMP-9 that co-localize with activated macrophages[4]. Numerous plaque components activate macrophages, including cytokines[5], oxidized LDL[5], C-reactive protein[6] (CRP), and immune complexes[7] (IC). Despite the fact that IC are present in plaques and activate macrophages through Fcγ receptor (FcγR) cross-linking, it is only now beginning to be recognized that IC can influence plaque progression though FcγR signaling. The generation of atherosclerosis prone (ApoE-/- and LDLR-/-) FcγR -/- double knockout mice enabled the role for FcγR in atherosclerosis to be studied in a mouse model. When fed a high fat diet, plaque area is significantly higher in single vs double knock-outs [8]–[9], implicating FcγR in atherogenesis. However, the link between FcγR activation and plaque vulnerability in humans is understudied. We postulate that plaque macrophages take up IC and secrete MMPs for matrix degradation. Herein we present evidence that vulnerable regions of human carotid plaques have a pattern of gene expression significantly different from paired proximal regions that are histologically stable. Additionally, we demonstrate that engagement of macrophage FcγRs with IC induces a pattern of gene expression similar to that of the vulnerable plaques, suggesting that FcγR-activated macrophages may play a greater role in plaque vulnerability than previously appreciated.

## Methods

### Patient selection criteria

This investigation conforms to the principles outlined in the Declaration of Helsinki. Specimens were procured and processed according to approved IRB protocols. The study is classified as exempt category 4; no informed consent was necessary. Femoral and carotid endarterectomy tissue was provided by The Vascular Group, PLLC based at Albany Medical Center. Femoral plaques (n = 14) were extracted by longitudinal arteriotomy, usually as an adjunct to distal bypass; carotids (n = 16) were removed using the eversion method from symptomatic patients (3/16 or 19%) and asymptomatic patients (13/16 or 81%) with >60% stenosis. Tissue was collected sequentially over a 6-month period with the only criteria for exclusion being absence of a morphologically distinct distal and proximal segment, making it an inherently randomized study.

### Tissue Collection and Quantitative RT-PCR

Immediately upon removal, the tissue was placed in RNAlater® (Qiagen, Inc. Valencia, CA) and transported to the lab for processing. Carotid plaques were divided into proximal (at, or near, the carotid bifurcation) and distal (up the internal carotid artery). From each carotid region, two contiguous blocks were taken, one for paraffin embedding and one preserved in OCT (optimal cutting temperature) for immunohistochemistry. RNA was extracted from the remainder of the tissue. Proximal and distal carotid and femoral tissue was weighed and RNA extracted with Tri-reagent (Molecular Research Center, Cincinnati, OH). Extraction was facilitated by homogenization with a Brinkman Polytron®. cDNA was prepared from RNA using iScript (Bio-Rad, Hercules, CA). qPCR primers (Table S1) were designed to amplify 100–200 bp fragments across an intron and were unique as determined by BLAST analysis. Relative abundance of mRNA was normalized to β-actin and calculated as 2^-(Ct gene – Ct actin)^ where Ct represents the threshold cycle for each transcript. The limits of detection were set at Ct ≥35. As the amount of RNA recovered varied with the specimen, the genes were prioritized (MMP, FcγR>MMP activators, inhibitors, signaling genes). Thus, the number of samples varies among the genes (n = 8–16).

**Table 1:** General characteristics of patient pool.

	Endarterectomy site (n = 30)		
*Characteristics* *	*Carotid*	*Femoral*	*p*
Total	16 (53.3)	14 (46.7)	
Age, (yrs)	67.8±9.1	65.8±8.7	.54
Male	10 (62.5)	10 (71.4)	.71
Risk Factors			
Hypertension	12 (75.0)	10 (71.4)	1.0
Hyperlipidemia	16 (100)	5 (35.7)	<.05^$^
Diabetes	2 (12.5)	5 (35.7)	.20
Cardiac Disease	9 (56.3)	8 (57.1)	1.0
Smoking	4 (25.0)	4 (28.6)	1.0
Symptomatic Carotid Disease	3 (18.8)		
Asymptomatic Carotid Disease	13 (81.3)		
Symptomatic Femoral Disease			
Claudication		7 (50.0)	
Rest Pain		2 (14.3)	
Non-Healing Ulcer		4 (28.6)	
Other		1 (7.1)	

*Data are presented as mean ± SD (%).

$ Statistically Significant.

### Histology

Paraffin-embedded tissue was sectioned (7 µm), stained with Tri-chrome, and classified using the American Heart Association stability criteria[2]. OCT-embedded tissue was cryosectioned (7 µm) and mounted on Superfrost slides. Fixation (acetone, 10 min) and staining was done at room temperature. Sections were blocked (45 min, PBS containing 1% Tween-20 and 10% horse serum) and primary antibodies incubated for 1 h. Biotinylated secondary antibodies (1∶200, 1 h) were visualized using the Vectastain ABC system (Vector Laboratories, Burlingame, CA). Primary antibodies were mouse monoclonals (Table S2); isotype controls (25 mg/ml) had no detectable staining. Immunohistochemistry was done on 3–4 different proximal plaques. Despite variations in plaque morphology (i.e., percent stenosis, eccentricity, distribution of fibrous tissue), the localization of proteins with respect to macrophages and smooth muscle cells was reproducible.

### Zymography

Tissue lysates were prepared by homogenization and sonication in RIPA buffer (50 mM Tris, pH 7.4, 150 mM NaCl, 12.7 mM deoxycholate, 25 mM glycerophosphate, 1% SDS, and 1% Triton X-100 containing protease inhibitors) followed by ultracentrifugation (1 h x 100,000 x g). 3 µg protein/well was run on 10% gelatin gels according to manufacturers' directions (Invitrogen, Carlsbad, CA); 20 µg of HT15 media was used as the positive control [10].

### Macrophage stimulation

Human monocytes, purified from peripheral blood by counter-flow centrifugal elutriation, were purchased from The University of Nebraska Medical Center. Elutriation separates the monocytes from other blood cells based on their sedimentation in a centrifugal field. The process uses physiological media, thus maintaining cell viability and function. Monocytes were differentiated (200 ng/ml recombinant human M-CSF, Cell Sciences, Canton, MA) for 7 days. THP-1s, a human macrophage-like cell line (ATCC #TIB-202), were differentiated with PMA (8.1 µM, 18 h) before use. Monocytes-derived macrophages (5×10^6^) and differentiated THP-1 cells (1×10^6^) were stimulated (18 h) with 200 ng/ml human TNF-α (Cell Sciences, Canton MA), 50 µg protein/ml oxLDL (Invitrogen, Carlsbad, CA), 40 µg/ml C-reactive protein (Calbiochem, San Diego, CA). The concentrations used reflect serum concentrations in patients with atherosclerosis [6], [11]. LDL was oxidized with 5 µM CuSO_4_ (20 h, 37°C); oxidation was confirmed using the TBARS assay with malondialdehyde as the standard [12]. Average level of oxidation was 18–23 nmol malondialdehyde equivalents/mg protein. Immune complexes (IC) were presented as IgG-opsonized 2 µm beads; 10 beads/cell reflecting standard phagocytosis ratios [13]. Following stimulation, RNA was extracted and qPCR performed as above.

### Statistical Analysis

The characteristics of the patient population were compared using unpaired Student's T-test and Fisher's Exact Test. For the gene analysis, the most appropriate statistical analyses were performed. Specifically, as the tissue came from different patients, gene expression in femoral and stable carotid specimens was compared using an unpaired Student's T-test. In contrast, the vulnerable and stable carotid tissue came from the same plaque. Thus, gene expression in these paired samples was compared using a paired Student's T-test. Within a gene, the tests were corrected for multiple comparisons using Bonferroni's correction and significance accepted at the 0.05 level. For MMPs, uPA, and uPAR, a logarithmic transformation was applied prior to significance testing to correct for heteroscadisicity. As expected, there is variability among the specimens. Although the extreme outlyers may be due to unknown complications in the patient, none were excluded. With a minimum of 8 patients, we estimate that we would have adequate power (>80%) for detection of differences between stable to vulnerable that are as large or larger than 120% of within-group standard deviation.

## Results and Discussion

Full demographic details of the 30 patients in the study are summarized in Table 1. No statistically significant differences were found between the carotid and femoral patient pools with regards to age, sex, hypertension, smoking, diabetes, and preexisting coronary artery disease; hyperlipidemia was higher in carotid vs femoral patient pools.

### Stable plaques, regardless of anatomical location, have similar gene expression patterns

**Figure 1:**
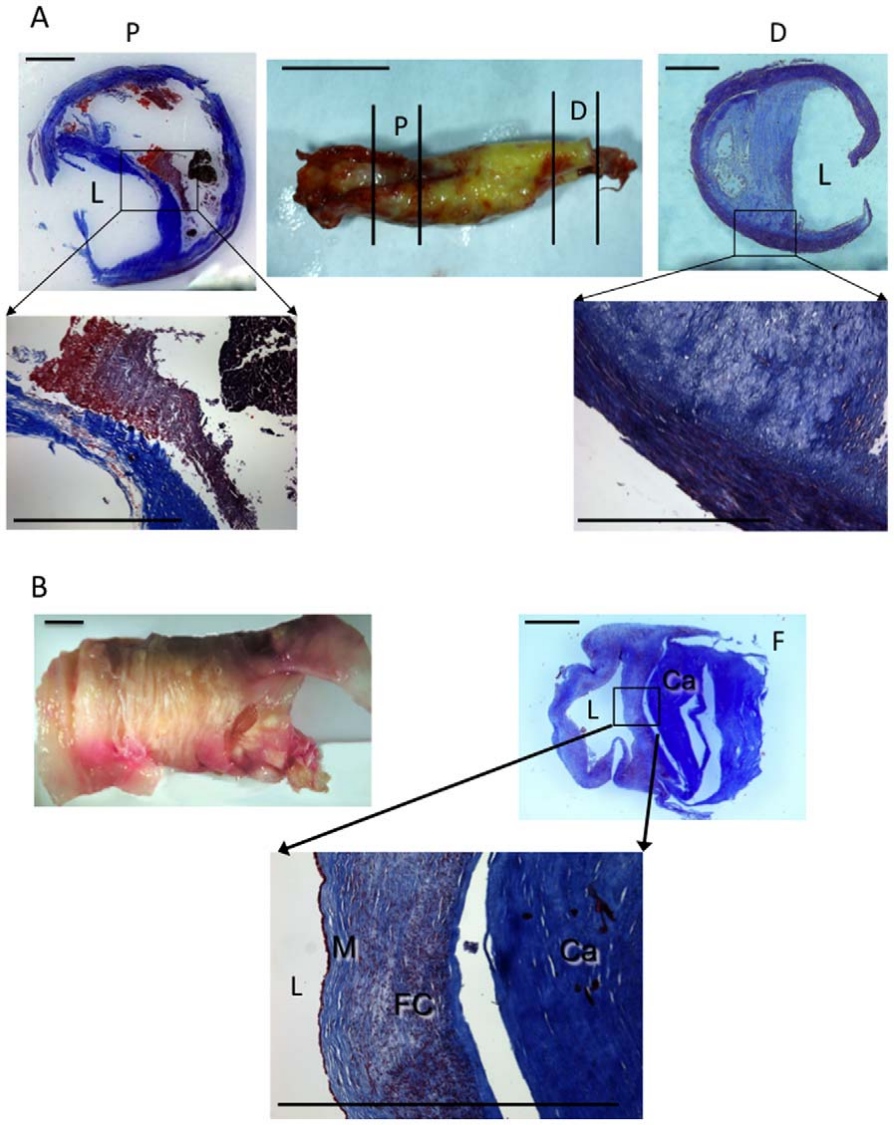
Histology of human carotid and femoral plaques. **(A)** Carotid endarterectomy specimen with proximal (P) and distal (D) regions indicated (bar  = 1 cm). Tri-chrome staining of the proximal (top, left) and distal (top, right) regions reveals necrotic cores with a thin cap compromised by cellular infiltration (bottom, left) or protected by a thick fibrous cap (bottom, right), respectively. Bars  = 1 mm. (B) Femoral plaques are characterized by an organized matrix (M) overlying a fibrocellular (FC) band deposited on a calcified (Ca) deposit. The lumen is designated with an L. Bar  = 1 mm.

To mitigate against stroke, carotid endarterectomy of asymptomatic plaques with ≥60% stenosis is recommended[14], [15], [16]. Carotid endarterectomy specimens are often visually and hsitologically heterogeneous (Figure 1A). While a necrotic/lipid core is present throughout, the core of the distal (relative to the heart) region was relatively small and protected by a thick fibrous cap while there was little or no cap overlying the proximal core (Figure 1A). In contrast, proximal plaque regions exhibited multiple histological indicators of instability (i.e., thin cap, intraplaque hemorrhage, large necrotic core). This heterogeneity is of considerable etiological interest. Is it possible that the fibrous regions represent an earlier stage in plaque development, with expansion of the fatty/necrotic core and activation of matrix degradation a subsequent event leading to plaque vulnerability. Alternatively, plaque histology may vary as a function of blood flow, with that in the internal carotid (distal) more laminar and that in the common carotid (proximal) being more disturbed. As human specimens represent an endpoint in the disease process, such etiological questions must be answered in animal models.

**Figure 2:**
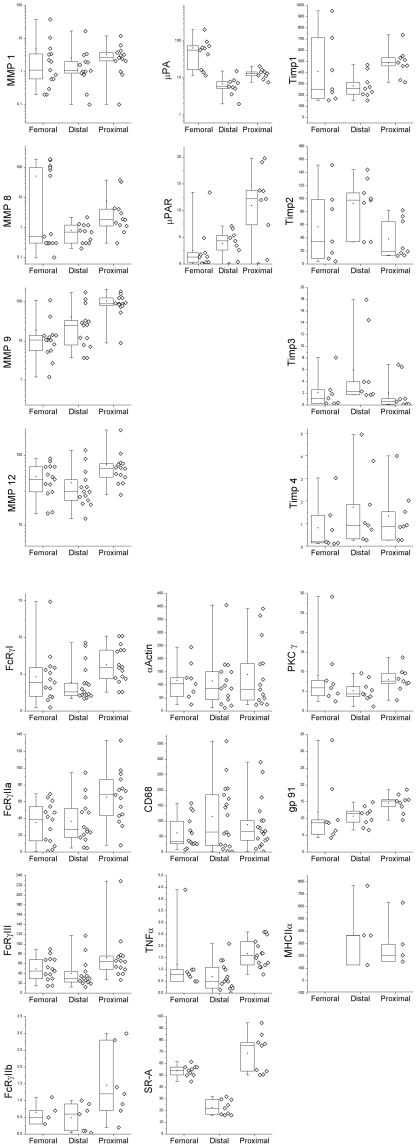
Expression levels for genes implicated in carotid plaque vulnerability. Distal carotid plaque tissue and femoral tissue have similar gene expression patterns; proximal and distal carotid tissue have significantly different levels of these genes. The relative mRNA expression for each gene for each plaque is represented by a single point. For each gene and tissue, a Box plot is shown to the left of the actual data. The Box plot indicates the median by a horizontal line within the box; the top and bottom of the box represent the 75^th^ and 25^th^ percentile, respectively; the whiskers indicate the range of the data and the dot indicates the mean. Statistical significance (p<0.05) is indicated by an asterisk (*) for proximal vs. distal and by a dagger (†) for femoral vs. distal. Expression was compared for femoral and distal carotid specimens using an unpaired Student's t-test.

In contrast to their carotid counterparts, femoral plaques rarely cause symptoms and thus are considered clinically stable, being removed or bypassed to relieve lower limb ischemia. Trichrome staining revealed that both femoral and distal carotid plaque tissue was fibrocellular, with cells (red) embedded in an extensive extracellular matrix (blue) (Figure 1, n = 14 and 16, respectively). Thus, we tested the hypothesis that their gene expression pattern was similar. Of particular interest were genes for macrophages (CD68) and smooth muscle cells (α-actin), matrix degradation (MMPs, TIMPs, uPA, and uPAR), macrophage activation (FcγRs, TNF-α, MHC II, gp91^phox^, PKC-δ, iNOS), the inhibitory FcγR IIb, and the scavenger receptor for oxLDL (SR-A). A qRT-PCR comparison between the femoral plaques and distal carotid plaque regions revealed no significant differences in expression of 20/22 genes (Figure 2); only SR-A and uPAR were significantly different (higher in femoral specimens).

SR-A is a scavenger receptor that internalizes oxLDL and contributes to foam cell formation. That it was higher in femoral plaques is intriguing as it suggests that elevated SR-A *per se* may not predispose a plaque to rupture. As soluble oxLDL is taken up by SR-A but doesn't generate a respiratory burst [17], SR-A may aid in the uptake of oxLDL by a mechanism that doesn't activate macrophages. Indeed, neither PKC-δ nor gp91^phox^, genes involved in the respiratory burst, were differentially expressed in femoral tissue (Figure 2B). Thus, elevated SR-A levels may be a physiological response to the increased circulating levels of oxLDL in patients with cardiovascular disease [18].

**Figure 3:**
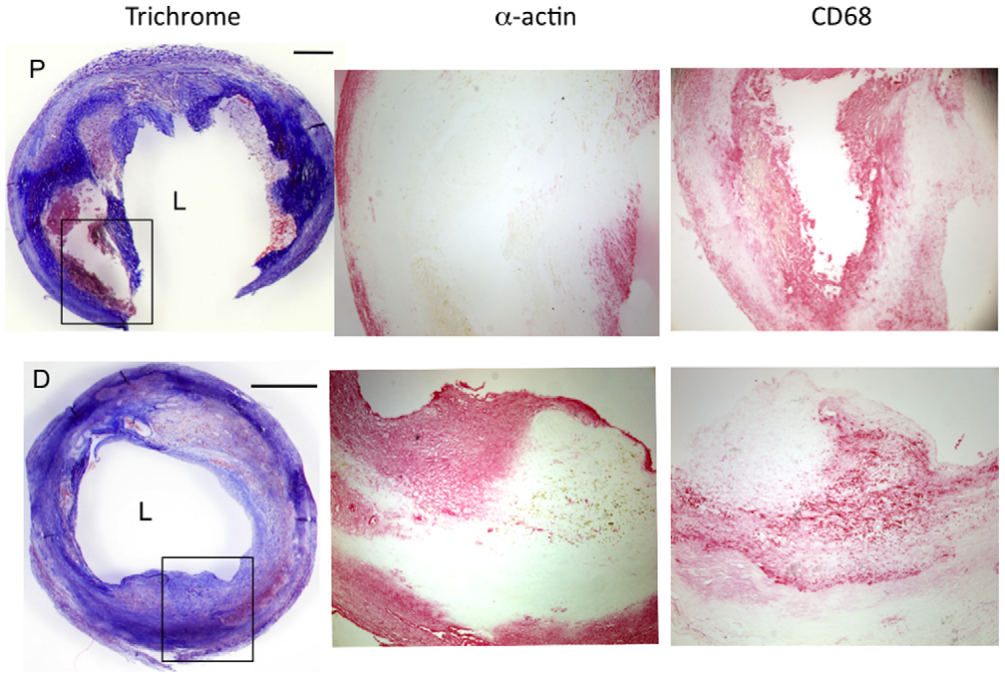
Macrophages and smooth muscle cells localize to discrete regions in both the proximal and distal regions of carotid plaques. The proximal (P) and distal (D) regions of a carotid plaque were stained with Trichrome to visualize fibrous tissue (blue) and cellular material (red). Smooth muscle cells and macrophages were visualized by immunohistochemistry for α-actin (smooth muscle cells) and CD68 (macrophages). Smooth muscle cells co-localize with the fibrous material while macrophages localize to acellular areas.

Most carotid plaques were visually heterogeneous, raising the question as to the differences in the regions (Figure 1A). That the distal region of carotid plaques had a gene expression pattern similar to clinically stable femoral plaques, allowed us to use the more powerful paired analysis to compare gene expression in the proximal and distal portions *of the same carotid plaque*. As the proximal portions of our carotid specimens had large necrotic cores, thin or no fibrous caps, and evidence of hemociterin laden foam cells (indicative of vulnerability), we predicted that they would contain more macrophages. Surprisingly, there was no significant difference in the expression of CD68 (macrophages) and α-actin (smooth muscle cells) between proximal and distal regions of carotid plaques (Figure 2). When stained for protein, CD68 and α-actin did not overlap (Figure 3). Thus, the differences in the amount of matrix (blue staining in Figure 1) could not be explained by the separation of collagen-producing smooth muscle cells from macrophages. Rather, the activation state of the macrophage may account for the decreased matrix in the proximal carotid plaques.

### Gene expression in proximal regions favors matrix degradation

**Figure 4:**
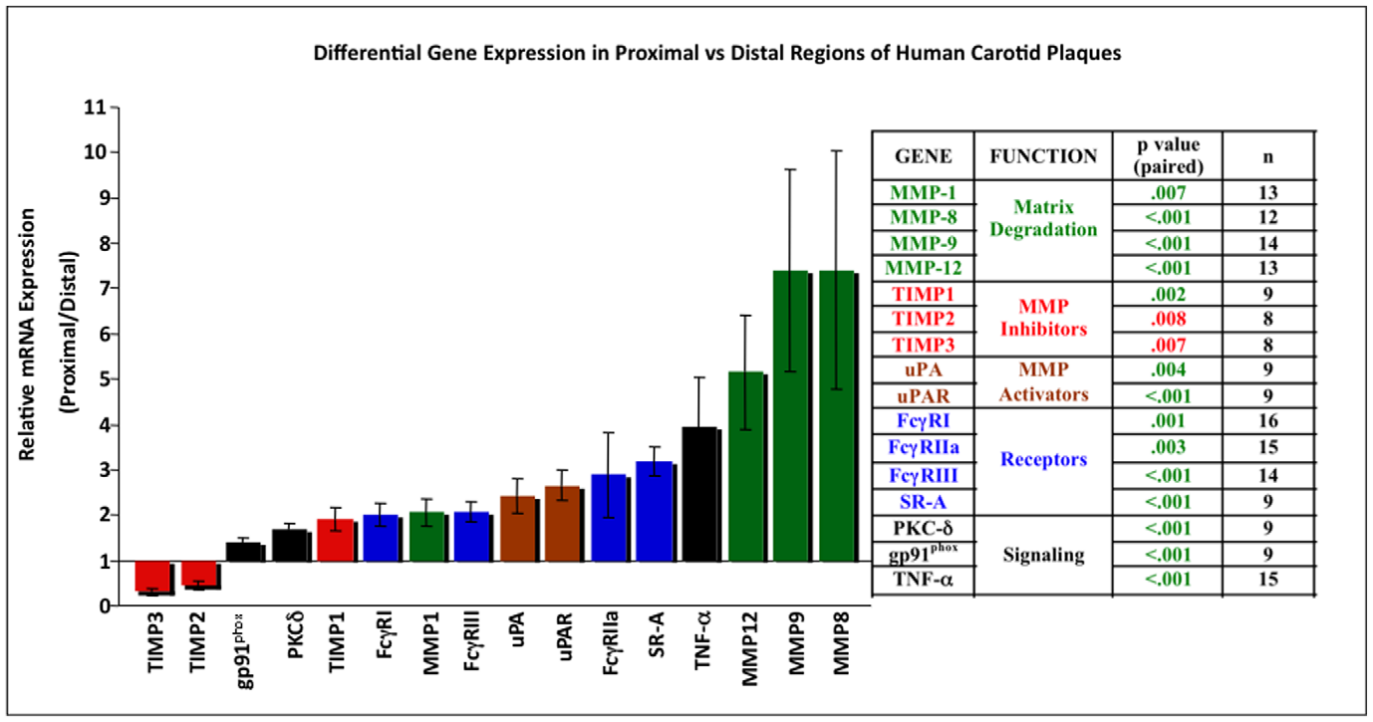
Relative gene expression in proximal vs distal plaque regions. A proximal/distal ratio was calculated from the qRT-PCR data generated for each paired sample in Figure 2. Results are presented as mean ± SEM (n = 8–16). Genes with a ratio >1 are more highly expressed in proximal tissue; ratios <1 indicate a decrease in gene expression in the proximal vs distal regions. Genes are grouped by color according to function. Statistical analysis and number of samples are presented in the accompanying table. Only genes that were significantly different (Figure 2) are presented.

When gene expression was compared between proximal and distal regions of the same carotid plaque, 17 genes were differentially expressed (Figure 4).

#### MMPs

**Figure 5:**
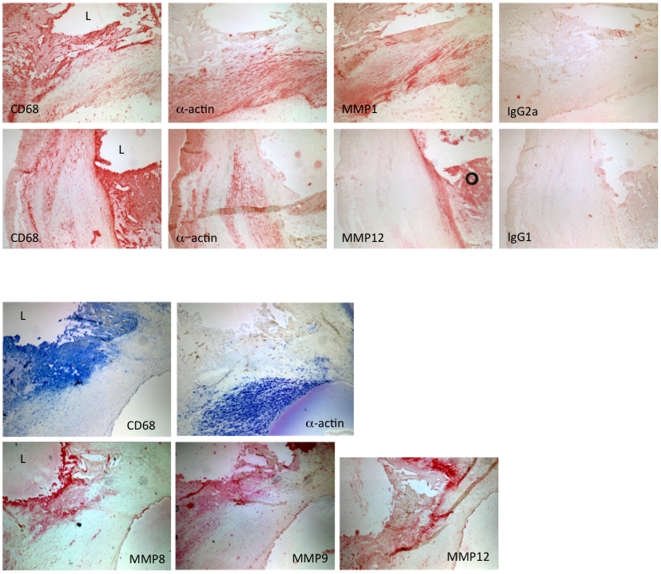
Matrix metalloproteinases localize to macrophages in proximal regions of carotid plaques. 7 µm cryosections were stained for macrophages (CD68) or smooth muscle cells (α-actin) by immunohistochemistry. CD68 and α-actin localized to discrete regions of the plaque. Sequential sections were stained for the indicated MMPs. MMP 8, 9, and 12 localized predominantly to the macrophages; MMP1 was present in both smooth muscle cells and macrophages. L marks plaque lumen. Representative of three plaques giving similar results.

**Figure 6:**
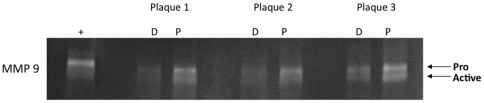
Proximal regions of carotid plaques contain more MMP-9 than corresponding distal regions. Distal (D) and proximal (P) regions were isolated from three carotid plaques and MMP9 levels were assessed by zymography using 3 µg of protein from each sample lysate. Proximal regions contained more MMP-9 protein of both the pro and active forms, paralleling the mRNA expression (Figure 2) and consistent with regulation of MMP-9 expression at the transcriptional level. +:20 µg of HT15 media was loaded as a positive control [10].

Proximal carotid tissue had higher MMP-1, -8, -9 and -12 mRNA expression compared to their corresponding distal regions (Figures 2,4). Immunohistochemistry revealed that MMP-8, -9 and -12 *protein* co-localized with macrophages in proximal regions; MMP-1 was predominantly associated with smooth muscle cells (Figure 5). Gelatin zymography was used to determine relative levels of MMP-9 protein in stable and vulnerable tissue; Figure 6 presents the results of gelatin zymography on 3 different carotid plaques. In all cases the proximal plaque regions contained more MMP-9/unit protein when compared to the corresponding distal segments. Thus, although not all MMP protein levels were examined, the fact that 1) MMPs are constitutively secreted by plaque macrophages [19], 2) they co-localized with macrophages (Figure 5), and that 3) mRNA for multiple MMPs is elevated in proximal carotid plaque tissue, is consistent with a model in which macrophages in proximal carotid plaques release MMPs that degrade the fibrous cap and destabilize the plaque. Although this concept is supported by published studies, this work is unique in that a comparison of gene expression has been done in stable and vulnerable regions of the same carotid plaque and has been combined with other markers of MMP regulation and macrophage activation.

#### uPA and uPAR

The uPA/uPAR system processes MMPs to their active forms. Accordingly, uPA and uPAR mRNA levels were significantly higher in proximal regions (Figures 2, 4). The combination of elevated uPA, uPAR, and MMPs favors MMP activation and extracellular matrix breakdown.

#### Tissue inhibitors of metalloproteinases (TIMPs)

**Figure 7:**
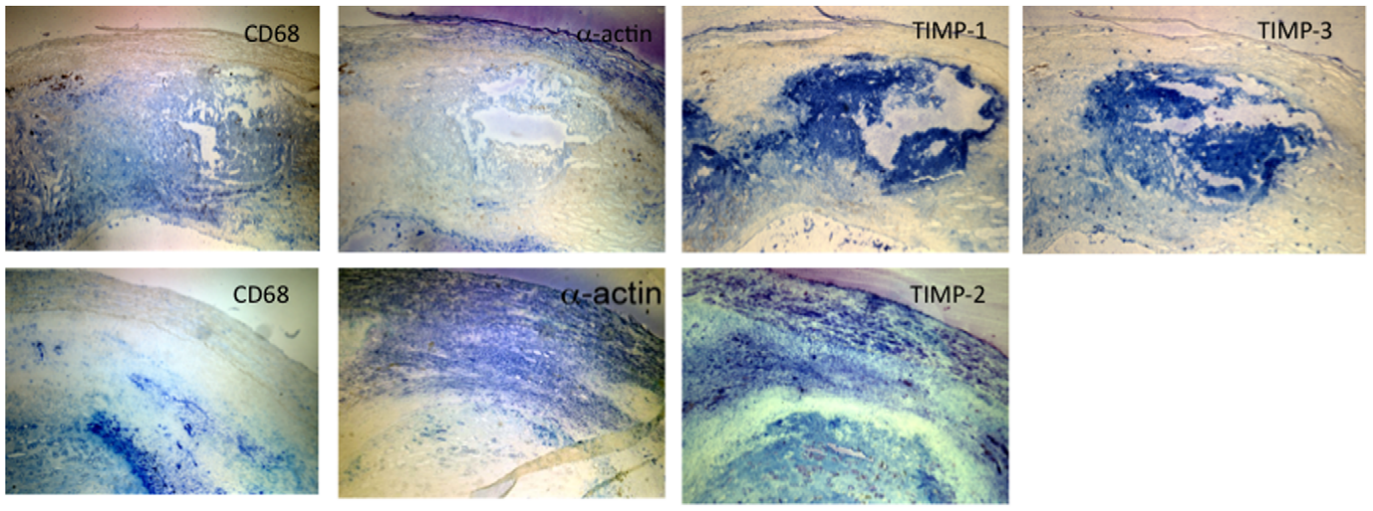
Localization of tissue inhibitor of metalloproteinases (TIMPs) in proximal carotid plaque tissue. 7 µm cryosections of carotid plaques were stained for macrophages (CD68) or smooth muscle cells (α-actin). Sequential sections were stained for the indicated TIMPs as described in Methods. Isotype controls are presented in Figure 4. TIMPs 1 and 3 co-localized with macrophages; TIMP2 was expressed in both macrophages and smooth muscle cells. Representative of three plaques giving similar results.

TIMPs bind to, and inhibit the activity of, mature MMPs. TIMP-2 and-3 mRNA were lower in proximal, compared to distal, regions (Figures 2, 4). TIMP-2 *protein* was distributed throughout the macrophage and smooth muscle cell regions while TIMP-3 localized to macrophages (Figure 7). Reduced TIMP-2 and-3 levels, combined with higher MMPs, would contribute to matrix degradation. TIMP-1 message was significantly higher in proximal vs distal regions (Figures 2, 4) and TIMP-1 protein localized to macrophages (Figure 7). This is consistent with reports that TIMP-1 overexpression does not alter plaque stability [20]. TIMP-4 mRNA was low and similar in both regions (Figure 2). This is not surprising as TIMP 4 is predominantly expressed in cardiac tissue.

Taken together, our results suggest that, compared with their distal counterparts, proximal carotid plaque regions contain higher levels of **active** MMP-9 (lower band, Figure 6) and MMP activating genes (uPA, uPAR, Figure 2). In these proximal regions, reduced TIMP-2 and-3, combined with higher MMPs, would favor matrix degradation (Figure 1), consistent with a phenotype more likely to rupture.

### Macrophages are activated in proximal regions of carotid plaques

Several subtypes of macrophages have been described, activated in different ways and expressing unique cadres of genes. Classically activated macrophages (M1 subtype) are pro-inflammatory, expressing elevated levels of Fc**γ**RI, Fc**γ**RIIa, Fc**γ**RIII, TNF-α, and releasing reactive oxygen species (ie, elevated NADPH oxidase) [21], [22]. In contrast, the M2 macrophages are immunoregulatory, with lower expression of FcγRI, FcγRIIa, FcγRIII (compared to M1), TNF-α, and reactive oxygen metabolites. As MMPs associate with macrophages in the proximal plaque regions (Figure 5), we evaluated the expression of genes associated with macrophage activation, including FcγRI, FcγRIIa, FcγRIII, TNF- α, MHCII, PKC-δ, and gp91^phox^; PKC-δ and gp91^phox^ were used as indicators of elevated NADPH oxidase. With the exception of MHCII, all of the genes were higher in proximal vs distal regions (Figure 2). This gene expression pattern is consistent with the presence of M1, classically activated macrophages [23] in the proximal plaque tissue. With their elevated expression of activating FcγR, proximal plaque macrophages would be primed for pro-inflammatory responses to immune complexes present in plaques.

In summary, the pattern of gene expression in proximal carotid plaque regions is consistent with activated macrophages (higher FcγR, TNF-α, gp91^phox^, PKC-δ) and increased MMP activity (i.e., higher MMP, uPA, and uPAR expression and lower TIMP 2,3) (Figure 4). These results define a cadre of genes, elevated in proximal plaque tissue, that can cooperate to increase the level of active MMPs, contributing to matrix degradation and increased plaque vulnerability.

### FcγR ligation in macrophages recapitulates the gene profile of proximal carotid plaques

**Figure 8:**
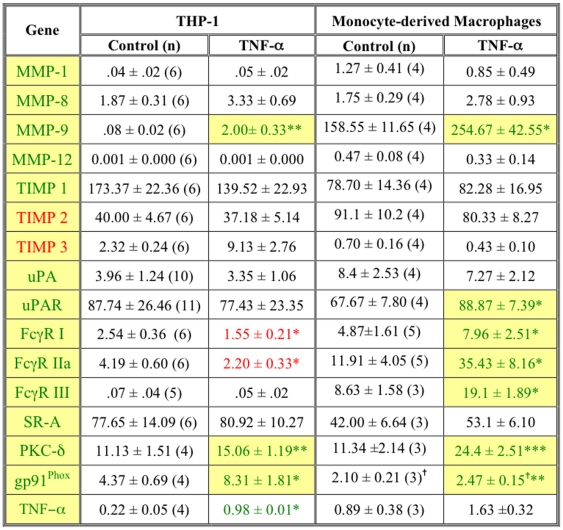
TNF-α does not induce a vulnerable gene profile in macrophages. Differentiated THP-1 cells or monocyte-derived macrophages were treated with 200 ng/ml human TNF-α for 18 h. RNA was extracted and subjected to qPCR. Data are presented as mean ± SEM (normalized to β-actin; n indicated in control column). Statistical significance compared by paired t-test for each replicate. * p<0.05, ** p<.01, *** p<.001. Yellow boxes denote genes similarly regulated in proximal vs distal plaque tissue. Green and red numbers indicate significantly increased or decreased expression, respectively compared to controls.^ = ^ Significance calculated from log values to compensate for heteroscedacity of the data.

**Figure 9:**
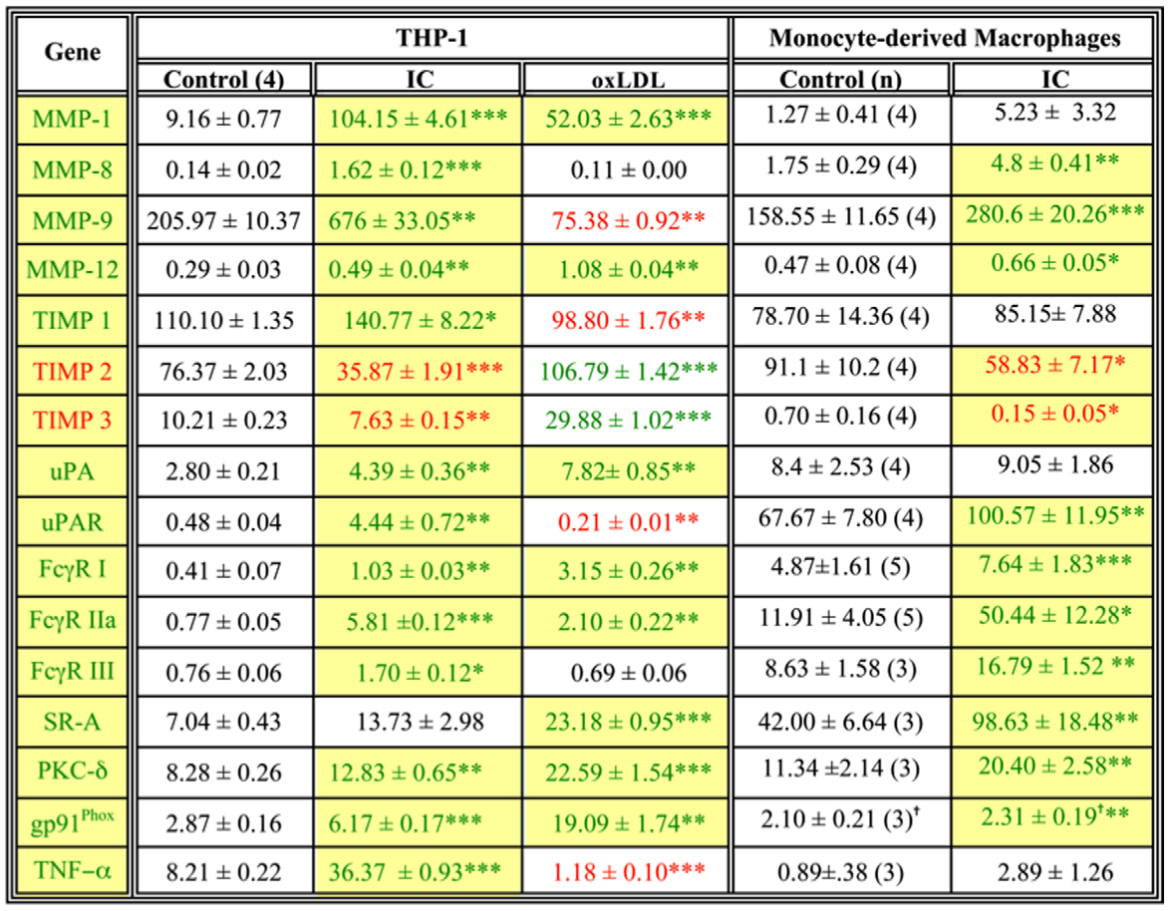
Immune complexes recapitulate the gene expression pattern of vulnerable plaques. Differentiated THP-1 cells or monocyte-derived macrophages were treated with immune complexes (10 beads/cell) or oxidized LDL (50 µg/ml). RNA was extracted and subjected to qPCR. Data are presented as mean ± SEM (normalized to β-actin; n indicated in control column). Statistical significance compared by paired t-test for each replicate. * p<0.05, ** p<.01, *** p<.001. Yellow boxes denote genes similarly regulated in proximal vs distal plaque tissue. Green and red numbers indicate significantly increased or decreased expression, respectively compared to controls. ^ = ^ Significance calculated from log values to compensate for heteroscedacity of the data.

Macrophages can be activated by many of the constituents of plaques, notably TNF-α, oxLDL, C-reactive protein (CRP), and immune complexes (IC). To assess the effects of macrophage activation on the expression of the genes associated with vulnerable carotid plaques, differentiated THP-1 cells and monocyte-derived macrophages were treated with TNF-α, oxLDL, CRP, or IC and the resulting changes in gene expression were quantified. Of the 16 genes differentially expressed in proximal plaques (Figure 4), TNF-α induced three in THP-1 cells and 7 in primary macrophages (Figure 8). Of the MMPs detected in proximal plaques, only MMP-9 was induced by TNF-α; TIMP levels were unaffected (Figure 8). CRP did not alter the expression of any of the genes (data not shown). Although oxLDL induced expression of 8 genes (Figure 9), several genes involved in matrix degradation (eg, MMP-9, TIMP-1, uPAR) were *decreased*, arguing against SR-A/oxLDL signaling as a major contributor to matrix degradation. This finding is consistent with our results demonstrating increased expression of SR-A mRNA in femoral plaques. Although SR-A is increased, its ligation with oxLDL may not induce MMP production and the plaques remain fibrous.

Of the four compounds tested, IC induced a pattern of gene expression most closely resembling that of proximal carotid plaque tissue. Fifteen of the 16 genes were similarly regulated in IC-treated THP-1 cells, 12 of 16 in primary macrophages (Figure 9). Of particular relevance to plaque vulnerability is that IC up-regulated the MMPs and down-regulated TIMPs 2 and 3. Thus, IC, but not TNF-α, oxLDL, or CRP, induced a pattern of gene expression in macrophages that recapitulates expression in vulnerable regions of carotid plaques, consistent with a model in which FcγR-dependent signaling contributes to plaque vulnerability.

If FcγR ligation contributes to an inflammatory environment, conditions that increase FcγR expression and/or signaling would be expected to be pro-atherosclerotic. Indeed, monocytes from patients with acute coronary syndrome have significantly higher FcγR expression [24]. Also, individuals expressing the R131 allele of FcγR IIa have increased peripheral atherosclerosis compared to those expressing H131[25]. Several other studies are consistent with a model in which FcγR signaling contributes to atherosclerosis. First, lovastatin inhibits FcγR-mediated phagocytosis[26]. Lower phagocytosis translates to less inflammation, a property of the drug that may contribute to its' atherosclerosis-reducing effects. Secondly, the double TNF-α/Apo E knockout mouse has decreased atherosclerosis when compared to the Apo E -/- single knockout[27]. Thus, TNF-α, which is rapidly produced upon FcγR ligation, and is up-regulated in vulnerable carotid plaques (Figures 2, 4), is pro-atherosclerotic. Finally, and most directly relevant, Apo E-/- mice lacking FcγRI and FcγRIII, and LDLR-/- mice deficient in FcγRIII have significantly less atherosclerosis than their Apo E/LDLR single knockout counterparts [8]–[9]. Conversely, deletion of the inhibitory FcγR IIb in Apo E-/- or LDLR -/- mice promotes atherosclerosis in the descending artery or aortic root [28], [29]. Our results provide a potential mechanism by which activating FcγR may contribute to matrix degradation and plaque vulnerability.

Admittedly, as macrophages in the plaque milieu are exposed to multiple stimuli this is an assessment focusing on the FcγR → MMP axis. Indeed, recent studies suggest that IC in conjunction with toll-like receptor ligation, may generate a protective response (review, [21]). However, the up-regulation of the activating FcγR, TNF-α, and gp91^phox^ suggest that the proximal carotid plaque environment is conducive to M1 (inflammatory) macrophage polarization. Laser capture microdissection will enable macrophages to be recovered from plaques. Expression profiling of such macrophages will permit a comprehensive comparison of genes differentially expressed in macrophages from vulnerable and stable plaques and their comparison to the profile generated by activation of macrophages through the FcγR. However, the results presented herein raise the intriguing possibility that FcγR ligation of plaque macrophages contributes to plaque instability, a model that, to date, has received little attention.

## Supporting Information

Table S1 **Sequence of qRT-PCR primers used in these studies.**
(DOC)

Table S2 **Antibodies used in these studies.** The isotype, company, and concentration used are listed. Note that Anti-MMP8, Anti-TIMP2, and control IgG 2a were used at 25 mg/ml.
(DOC)
