# Supplementary material for: Ligation of Macrophage Fcγ Receptors Recapitulates the Gene Expression Pattern of Vulnerable Human Carotid Plaques
Source: PLoS One. 2011 Jul 21;6(7):e21803. doi: 10.1371/journal.pone.0021803 (PMC3140977; doi:10.1371/journal.pone.0021803)
Supplement: Table S1 — Sequence of qRT-PCR primers used in these studies. (DOC) [file pone.0021803.s001.doc]

**Table S1. qPCR Primers**

| **GENE** | **SEQ** | **PRIMER SEQUENCE 5’ TO 3’** |
| --- | --- | --- |
| **β-actin** | Forward | TACCTCATGAAGATCCTCACC |
|  | Reverse | TTTCGTGGATGCCACAGGAC |
|  | | |
| **MMP-1** | Forward | TGCAACTCTGACGTTGATCCCA |
|  | Reverse | cggcaaagactcatgtctcctgTc |
|  | | |
| **MMP-8** | Forward | AACCCCAGGTACCCATGAATGT |
|  | Reverse | CCCACCCTAATTCAGTGTGATCTC |
|  | | |
| **MMP-9** | Forward | AGGAGCCAGTTTGCCGGAT |
|  | Reverse | AGGTGAGAAGAGAGGGCCCA |
|  | | |
| **MMP-12** | Forward | AGTTACCTTCAAAGGCCAAGAG |
|  | Reverse | TCCAAGGATGTTAGGAAGCAAC |
|  | | |
| **TIMP-1** | Forward | GTCATCAGGGCCAAGTTCG |
|  | Reverse | TGAGAAACTCCTCGCTGCG |
|  | | |
| **TIMP-2** | Forward | AAGCGGTCAGTGAGAAGGAAG |
|  | Reverse | CACACACTACCGAGGAGGG |
|  | | |
| **TIMP-3** | Forward | TGCAACTTCGTGGAGAGGTG |
|  | Reverse | CACAAAGCAAGGCAGGTAGTA |
|  | | |
| **TIMP-4** | Forward | AGTATCTCTTGACTGGTCAGGTC |
|  | Reverse | GCAGCCACAGTTCAGATGGTA |
|  | | |
| **FcγRI** | Forward | ATGTGGTTCTTGACAACTCTG |
|  | Reverse | GTAGCTGGGGGTCGAGGT |
|  | | |
| **FcγRIIa** | Forward | ATGACTATGGAGACCCAAATG |
|  | Reverse | ATTGTGGAACCACTGAATGGA |
|  | | |
| **FcγRIIb** | Forward | CTCTCCCAGGATACCCTGAGT |
|  | Reverse | GGTGCATGAGAAGTGAATAGGTG |
|  | | |
| **FcγRIII** | Forward | ATGTGGCAGCTGCTCCTC |
|  | Reverse | CGAGGCCTGGCTTGAGAT |
|  | | |
| **uPA** | Forward | TTCGGAGGGCAGCACTGTGA |
|  | Reverse | ATGTTTCCCCAGGCCCAGCT |
|  | | |
| **uPAR** | Forward | GGGGATTGCCGTGTGGAAGA |
|  | Reverse | ATTGATTCATGGGGCCTCGG |
| **GENE** | **SEQ** | **PRIMER SEQUENCE 5’ TO 3’** |
| **PKCδ** | Forward | ATGGCGCCGTTCCTGCGC |
|  | Reverse | TAGCACAATCTGGATGACGC |
|  | | |
| **gp91^Phox^** | Forward | CTGGTTTGGCTGGGGTTG |
|  | Reverse | CCTGAGGAAGGACAGCAG |
|  | | |
| **α-Actin** | Forward | GGCTTTGCTGGGGACGAT |
|  | Reverse | AGGGGCAACACGAAGCTC |
|  | | |
| **CD68** | Forward | GCTGTGCTTTTCTCGGGG |
|  | Reverse | AGTGGCAGTCGTGGGTCC |
|  | | |
| **TNF-α** | Forward | TCTCGAACCCCGAGTGAC |
|  | Reverse | GGCAGCCTTGGCCCTTGA |
|  | | |
| **iNOS** | Forward | ACCTTCAGTATCACAACCTCA |
|  | Reverse | GACCTGCAAGTTAAAATCCCT |
|  | | |
| **SR-A1** | Forward | CGGGAAAAGGAAATGACAGCG |
|  | Reverse | ATT GCA TTC CCA TGT CCC TGG |
|  | | |
| **MHC II** | Forward | GACCAATCAGGCGAGTTTATGT |
|  | Reverse | TGAGCACAGTTACCTCTGGAG |
