# Supplementary material for: Ligation of Macrophage Fcγ Receptors Recapitulates the Gene Expression Pattern of Vulnerable Human Carotid Plaques
Source: PLoS One. 2011 Jul 21;6(7):e21803. doi: 10.1371/journal.pone.0021803 (PMC3140977; doi:10.1371/journal.pone.0021803)
Supplement: Table S2 — Antibodies used in these studies. The isotype, company, and concentration used are listed. Note that Anti-MMP8, Anti-TIMP2, and control IgG 2a were used at 25 mg/ml. (DOC) [file pone.0021803.s002.doc]

**Table S2. Anitbodies and concentrations used for immunohistochemistry**

| **Mouse** | **Isotype** | **Company** | **Cat. No.** | **Concentration** |
| --- | --- | --- | --- | --- |
| Anti-MMP1 | IgG2a | Calbiochem | 1M35 | 25 µg/ml |
| Anti-MMP8 | IgG2a | R&D Systems | MAB9081 | 25 mg/ml |
| Anti-MMP9 | IgG1 | R&D Systems | MAB936 | 15 µg/ml |
| Anti-MMP12 | IgG1 | R&D Systems | MAB917 | 25 µg/ml |
| Anti-TIMP1 | IgG1 | Santa Cruz | SC-21734 | 25 µg/ml |
| Anti-TIMP2 | IgG2a | Santa Cruz | SC-21735 | 25 mg/ml |
| Anti-TIMP3 | IgG1 | R&D Systems | MAB973 | 25 µg/ml |
| Anti-CD68 | IgG1 | Santa Cruz | SC-20060 | 5 mg/ml |
| Anti-α-actin | IgG2a | Santa Cruz | SC-32251 | 5 µg/ml |
| Control | IgG1 | Santa Cruz | SC-3877 | 25 µg/ml |
| Control | IgG2a | Santa Cruz | SC-3878 | 25 mg/ml |
